# Prediction of Antimicrobial Resistance in Gram-Negative Bacteria From Whole-Genome Sequencing Data

**DOI:** 10.3389/fmicb.2020.01013

**Published:** 2020-05-25

**Authors:** Pieter-Jan Van Camp, David B. Haslam, Aleksey Porollo

**Affiliations:** ^1^Department of Biomedical Informatics, University of Cincinnati, Cincinnati, OH, United States; ^2^Division of Biomedical Informatics, Cincinnati Children’s Hospital Medical Center, Cincinnati, OH, United States; ^3^Division of Infectious Diseases, Cincinnati Children’s Hospital Medical Center, Cincinnati, OH, United States; ^4^Department of Pediatrics, University of Cincinnati, Cincinnati, OH, United States; ^5^Center for Autoimmune Genomics and Etiology, Cincinnati Children’s Hospital Medical Center, Cincinnati, OH, United States

**Keywords:** antimicrobial resistance, antibiotic resistance, whole-genome sequencing, machine learning, prediction, genotype-phenotype relationship

## Abstract

**Background:**

Early detection of antimicrobial resistance in pathogens and prescription of more effective antibiotics is a fast-emerging need in clinical practice. High-throughput sequencing technology, such as whole genome sequencing (WGS), may have the capacity to rapidly guide the clinical decision-making process. The prediction of antimicrobial resistance in Gram-negative bacteria, often the cause of serious systemic infections, is more challenging as genotype-to-phenotype (drug resistance) relationship is more complex than for most Gram-positive organisms.

**Methods and Findings:**

We have used NCBI BioSample database to train and cross-validate eight XGBoost-based machine learning models to predict drug resistance to cefepime, cefotaxime, ceftriaxone, ciprofloxacin, gentamicin, levofloxacin, meropenem, and tobramycin tested in *Acinetobacter baumannii*, *Escherichia coli*, *Enterobacter cloacae*, *Klebsiella aerogenes*, and *Klebsiella pneumoniae*. The input is the WGS data in terms of the coverage of known antibiotic resistance genes by shotgun sequencing reads. Models demonstrate high performance and robustness to class imbalanced datasets.

**Conclusion:**

Whole genome sequencing enables the prediction of antimicrobial resistance in Gram-negative bacteria. We present a tool that provides an *in silico* antibiogram for eight drugs. Predictions are accompanied with a reliability index that may further facilitate the decision making process. The demo version of the tool with pre-processed samples is available at https://vancampn.shinyapps.io/wgs2amr/. The stand-alone version of the predictor is available at https://github.com/pieterjanvc/wgs2amr/.

## Introduction

Since the discovery and widespread use of antibiotics (AB) in the early 20th century, resistance to those same AB has generally developed rapidly; often even within the first years of introduction (Marston et al., 2016). As a consequence, many bacteria have developed antibiotic resistance (ABR) to most of the major classes of AB, often seen in the Gram-negatives (Centers for Disease Control and Prevention, 2018). Effective treatment of these infections requires knowledge of the organism’s susceptibility to the various AB, currently obtained by culturing bacteria in the clinical laboratory and subsequent testing for commonly used AB. Depending on the pathogen, this process may require 72 h or more. Drug susceptibility is usually reported to the clinician as either resistant or susceptible (sometimes intermediate is also used) with cut-offs based on the minimum inhibitory concentration (MIC) of an AB needed to halt growth or kill the pathogen in the lab. In serious systemic infections, early treatment with an effective antibiotic is paramount as unexpected resistance may lead to treatment failure, while fear of inadequate therapy may drive overly broad antibiotic use which contributes to extensively resistant and potentially untreatable bacteria (Marston et al., 2016).

With decreasing cost and increasing speed of high-throughput sequencing technology such as whole genome sequencing (WGS), in-depth analysis of pathogens is increasingly used in clinical decision-making. Studies already showed the potential of these techniques in ABR prediction in single, Gram-positive pathogens like *Staphylococcus aureus* and *Mycobacterium tuberculosis*. An example is the Mykrobe predictor that maps DNA sequencing data to a reference genome and a set of plasmid genes conferring ABR (Bradley et al., 2015). The model also accounts for polymorphism in select loci when predicting drug resistance. Data analysis and prediction is rapid, enabling it as a practical tool for clinical care during the decision-making process. PhyResSE is another tool that follows a similar strategy but may process data in minutes to a few days, attributing such time extension to more careful variant calling (Feuerriegel et al., 2015). Both tools report high accuracy of ABR prediction. However, their application to other pathogens like Gram-negative bacteria has not been described.

The prediction of ABR in Gram-negative bacteria, often the cause of serious systemic infections, is more challenging as the source of drug resistance is more complicated. For example, Gram-negative pathogens may possess one or more β-lactamases with similar amino acid sequences but various activity against β-lactam-based AB (Livermore, 1998). They are also more likely to develop mutations that result in lower membrane permeability, or increase the expression of a variety of genes for excreting xenobiotics (efflux pumps) and for inactivating β-lactam-targeting drugs (Marston et al., 2016). There have been a few clinical predictors reported for assessing the risk of infection with resistant Gram-negative bacteria but they solely rely on data from the electronic health record and just predict the likelihood of infection with a resistant strain rather than individual drug resistance (Martin et al., 2013; Vasudevan et al., 2014). Thus far, only a handful of studies were published on the prediction of Gram-negative resistance using WGS data. These models were built for individual species, such as *Neisseria gonorrhoeae* or *Klebsiella pneumonia*, and were based on small sample sizes resulting in poor predictive accuracy (Eyre et al., 2017; Nguyen et al., 2018). A more extensive study recently published by Drouin et al. (2019) utilizes 107 machine-learning-based models, trained on WGS data, to predict ABR in 12 bacterial species, including six Gram-negatives, against a variety of AB with generally high accuracy. These models were trained without incorporating prior ABR knowledge as they were based on nucleotide k-mers from sequenced genomes of these pathogens, thus utilizing information from non-coding regions and polymorphism. Furthermore, their methodology produced small, human-interpretable decision trees, when the k-mers are mapped back to the respective genomes to make decisions interpretable (provided that k-mers belong to annotated genomic regions). However, the study was performed on heavily imbalanced datasets, while reporting only two-class accuracy, which could overestimate the real performance. Finally, isolates with intermediate resistance were excluded from the final predictors making the performance estimates on such samples uncertain. For more details on the bioinformatics approaches to the AMR analysis and prediction, the reader can refer to a recent review (Van Camp et al., 2020).

In this work, we present a machine learning-based method for the fast estimation of ABR in 5 Gram-negative species for 8 AB. The models were trained on WGS data and laboratory confirmed drug susceptibility. All isolates were assigned to either of two classes (susceptible or resistant) and subsequently used in the training and validation of binary predictors. Each model was evaluated using multiple performance measures. The presented workflow could inform early clinical decision-making on the choice of AB therapy (within a day) while waiting for the final antibiogram (typically 2–4 days) thereby decreasing the time to start effective therapy. A web-based demo application to show the potential clinical implementation is publicly available at https://vancampn.shinyapps.io/wgs2amr/. The stand-alone tool can be freely downloaded from https://github.com/pieterjanvc/wgs2amr/.

## Materials and Methods

### Pathogens and Antibiotics of Interest

This study focused on five common nosocomial Gram-negative organisms that can cause sepsis (Vincent, 2003; Couto et al., 2007): *Acinetobacter baumannii*, *Escherichia coli*, *Enterobacter cloacae*, *Klebsiella aerogenes*, and *Klebsiella pneumoniae*. These specific Gram-negative pathogens were chosen because, in addition to clinical relevance, they are the most represented in terms of DNA-sequenced and AMR annotated samples available at NCBI. For machine learning, the larger the dataset to train on, the more accurate and generalized model can be achieved. *E. cloacae* is the major representative of *Enterobacter* species and therefore part of ESKAPE pathogens. Their antibiotic susceptibility was evaluated for cefepime, cefotaxime, ceftriaxone, ciprofloxacin, gentamicin, levofloxacin, meropenem, and tobramycin. This panel of AB covers those most commonly used to treat Gram-negative bacterial infections. Consequently, these AB are most frequently tested for susceptibility against bacterial isolates.

### Public Data Collection

**FIGURE 1:**
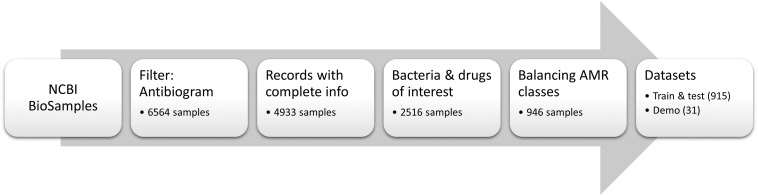
Data collection of public samples from NCBI. The numbers of samples represent total samples remaining in the dataset after a given data processing step.

Meta-data for 6564 bacterial samples (isolates) were retrieved from the NCBI BioSample database using the “antibiogram” keyword filter. Of these, 4933 samples had the required information, such as bacterium name, antibiogram, and sequencing data accession number. For this work, all “intermediate” ABR levels were converted to “resistant” to project data to a binary classification problem (i.e., resistant versus susceptible). The list was subsequently refined to only include the bacteria and AB of interest (see section “Pathogens and antibiotics of interest”) resulting in 2516 samples. Given the resistance to AB was highly imbalanced in the data (mostly skewed toward resistant phenotype), the samples were randomly chosen so that the number of susceptible, and resistant isolates for each antibiotic was as equal as possible in order to balance the input for machine learning models. This resulted in a final total of 946 samples (Supplementary Table S1). Of these, 3% of samples available for each species (total *n* = 31) were set aside to create a demo dataset to showcase the online application (see section “Preliminary pipeline implementation and evaluation” for details). The remaining 915 samples were used to build and evaluate eight XGBoost-based models, where available data for each antibiotic were randomly split in 70% training and 30% testing subsets. The overall flow of data collection is summarized in Figure 1. The counts of samples per species include: *A. baumannii* – 256; *E. cloacae* – 67; *E. coli* – 330; *K. aerogenes* – 51; and *K. pneumoniae* – 211. Of note, we did not stratify samples by different bacterial species during the model training as we intended our models to be species independent. Table 1 shows the distribution of the 915 samples through the AB of interest.

**TABLE 1:** Summary of the 915 samples used to build and evaluate antimicrobial resistance prediction models.

**Antibiotic**	**Resistant**	**Susceptible**	**Total**
Cefepime	442	275	717
Cefotaxime	437	50	487
Ceftriaxone	671	133	804
Ciprofloxacin	577	335	912
Gentamicin	351	542	893
Levofloxacin	471	258	729
Meropenem	332	420	752
Tobramycin	354	320	674

*Note that not all samples were tested for every antibiotic, thus the counts per AB do not add up to 915.

Whole genome sequencing data for all samples were retrieved from the NCBI Sequence Read Archive (SRA) using the SRA toolkit (SRA Toolkit Development Team, 2019). In case multiple runs (SRR) of a sample (SRS) were available (e.g., sample was run through different sequencers or with different settings), the file with the smallest size was selected. Smaller file size may frequently be attributed to lower sequencing depth and/or shorter shotgun reads. We reasoned that, if a model is able to make predictions on smaller sequence files, it will most likely be applicable to larger files with better gene coverage. List of all samples used can be found in Supplementary Table S1.

### In-House Dataset of Samples

Gram-negative bacterial pathogens were collected from blood or urine of patients hospitalized at Cincinnati Children’s Hospital Medical Center (CCHMC), 19 samples total. Organisms were identified to the species level and antimicrobial susceptibility testing was performed using the VITEK^®^ 2 machine (Biomerieux) in the Diagnostic Infectious Diseases Testing Laboratory at CCHMC. Samples represent *A. baumannii* (*n* = 1), *E. coli* (*n* = 11), *K. aerogenes* (*n* = 2), and *K. pneumoniae* (*n* = 3). No in-house samples are available with *E. cloacae*. Two available samples of *Klebsiella oxytoca* are included. DNA was extracted from overnight liquid broth cultures using the QIAamp PowerFecal DNA Kit (Qiagen Inc, Germantown, MD, United States). Sequencing libraries were generated using the Nextera XT kit (Illumina Corporation, San Diego, CA, United States). Pooled libraries were sequenced on a NextSeq 500 (Illumina Corporation, San Diego, CA, United States) in the Microbial Genomics and Metagenomics Laboratory at CCHMC using paired 150 bp reads to a depth of approximately 5 million reads per sample. Sample collection was approved by the Institutional Review Board (IRB) at CCHMC (IRB approval # 2016–9424: Molecular Epidemiology of Bacterial Infections). The in-house samples are available at the NCBI BioSample database (BioProject ID: PRJNA587095), where detailed metadata can be found (see Supplementary Table S1 for sample IDs). Of note, the antimicrobial susceptibility testing with VITEK takes at least 72 h and generally requires a pure isolate, whereas sequencing preparation followed by the WGS data analysis can be completed under 48 h and does need to not rely on a pure colony (Scaggs Huang et al., 2019).

### Analysis of WGS Data

The sequencing files in the FASTQ format were aligned to 4579 antibiotic resistance genes (ARG) found in the NCBI Bacterial Antimicrobial Resistance Reference Gene Database (NCBI Accession: PRJNA313047, version as of September 26, 2018) using DIAMOND (Buchfink et al., 2015), a greatly improved version of BLASTx (Altschul et al., 1990) with regards to speed, and on par sensitivity. Given the same ARG can be present in multiple species (e.g., plasmid DNA), no filtering was performed based on original bacterium in which the ARG was sequenced. The default settings of DIAMOND were used to map shotgun reads to ARGs.

Antibiotic resistance genes coverage (*C*) within a sample was quantified as follows. First, reads aligned by DIAMOND were discarded if the alignment to the gene covered <90% of the read’s length or yielded <90% sequence identity. Second, a gene was discarded if all aligned reads covered <90% of its length (i.e., protein sequence). The cutoff was chosen as being suboptimal after probing 70, 80, 90, and 100% coverage (Supplementary Table S2). If a gene was retained in the hit list, the number of alignments (*n*) was adjusted for gene length (i.e., the number of amino acids, *L*), and sequencing depth (i.e., total number of reads in the file divided by 10^7^, *D*). Such scaled data (Eq. 1) are better suitable for the machine learning.

(1) $$C=\frac{n}{L\times D}$$

### Clustering Similar Antibiotic Resistance Genes

Many of the ARG in the NCBI database have a high sequence similarity (e.g., polymorphism in strains or sequences derived from closely related species) that cannot be discriminated by the alignment techniques used in this study. Therefore, antibiotic resistance gene clusters (ARGC) were created using the cluster_fast module of the USEARCH algorithm (Edgar, 2010) to group genes with ≥90% sequence identity together, naming them after the most representative gene as defined by USEARCH (i.e., cluster centroid). This threshold for grouping was chosen as a suboptimal compromise between balancing the number of genes per cluster, performance of the model, and the biological relevance of the genes grouped together (Supplementary Table S3). For cutoffs lower 85%, genes from different ABR classes started to group together, hence such cutoffs were excluded from consideration. The clustering resulted in the reduction of the potential input space for the machine learning models from 4579 ARG to 1027 ARGC, with 410 clusters (40%) consisting of just a single gene. To represent the coverage of each ARGC, the average coverage (Eq. 1) of all ARG detected in this ARGC was taken. Finally, of the 1027 ARGC, only 152 were found in our data and thereby used for subsequent machine learning.

### Building and Evaluating Machine Learning Models

Regression models are less stable on datasets where the input space is large, sparse, and the features are correlated (e.g., in the context of this work, drug resistance may be exerted by multiple ARGC; Farrar and Glauber, 1967; Devika et al., 2016). Using penalized regression (e.g., LASSO and Ridge regression) to reduce both the input space and select most important features can help increase performance of the model, but it still operates on the premise that input features are uncorrelated. In correlated datasets, feature selection will be distorted in this process resulting in less reliable model interpretation. Decision trees, on the other hand, inherently perform better in such cases as correlation does not influence the feature selection process (Piramuthu, 2008). In random forest models, hundreds to thousands of these trees are built, each with different subset of the input space, resulting in a more robust reporting of important features. Neural networks (NN), especially their currently popular application to deep learning, and require much larger training data-sets (tens of thousands to millions of input vectors/samples) than currently available for antimicrobial resistance (hundreds of samples) in order to demonstrate benefits of deep learning. Moreover, the resulting NN-based models represent a black box that would be difficult to dissect in order to see the decision making rules and factors influencing the decision (Van Camp et al., 2020).

XGBoost is an extreme gradient booster for decision trees that is capable of handling correlated inputs. It has innate support for sparse datasets (in our case, only a handful of ARGC are present in each sample) and can extract important features to provide additional insights in the decision-making process (Chen and Guestrin, 2016). Although XGBoost supports multi-class classification (i.e., model can choose between more than two classes), our samples can have resistance to multiple AB at the same time (i.e., multi-labeling classification), which is not supported and thus a separate, independent binomial model (resistance versus susceptible) was created for each antibiotic of interest (8 models total).

The input for each model was the list of ARGC and their presence (*C* > 0) or absence (*C* = 0) in each sample (Eq. 1). The output was binomial with label *resistant* (= 1) or *susceptible* (= 0) to the antibiotic of interest. The XGBoost models were trained with a learning rate of 0.1, maximum tree depth of 2, training subsampling of 0.8, and column subsampling of 0.8. All other parameters were kept default. The algorithm was run for a maximum of 300 iterations, but early stopping was done when no improvement was seen in 50 consecutive iterations using 10-fold cross-validation.

Despite the efforts to balance the number of samples with susceptible and resistant phenotypes per drug the final distributions on individual AB remained unbalanced (Table 1) because each sample was not tested for all drugs of interest. For two most imbalanced drugs, cefotaxime and ceftriaxone, an under-sampling was performed to reach 3:1 ratio and prevent the model overfitting toward the over-represented class. For heavily class-imbalanced data, standard performance measures like sensitivity or 2-class accuracy could overestimate the true performance. The Matthew’s correlation coefficient (MCC) was therefore used as a more stringent performance statistic (Eq. 2). MCC measures binary classification in unbalanced datasets with range from -1 (inverse prediction) through 0 (random prediction) to 1 (perfect prediction).

(2) $$\text{MCC}=\frac{T\,P^{*}\,T\,N-F\,P^{*}\,F\,N}{\sqrt{\left(T\,P+F\,P\right)\,\left(T\,P+F\,N\right)\,\left(T\,N+F\,P\right)\,\left(T\,N+F\,N\right)}}$$

where *TP*, *TN*, *FP*, and *FN* are true positive, true negative, false positive, and false negative instances, respectively.

For complete performance evaluation, two-class accuracy (Acc, Eq. 3), sensitivity (recall, R), precision (P), specificity (Sp), areas under ROC (AUC), and precision-recall (PR-AUC) curves are also provided.

(3) $$A\,c\,c=\frac{T\,P+T\,N}{T\,P+F\,P+T\,N+F\,N}$$

where *Acc* is a two-class accuracy; *TP*, *TN*, *FP*, and *FN* are the same as in Eq. 2.

### Reliability Index

To provide an additional assessment how certain the prediction is by a given model, we introduce a reliability index (RI). The RI is based on the observation that in classification models values closer to extremes (0 or 1) are more likely to yield a correct prediction compared to values hovering around 0.5. Using adjusted model output (AMO, Eq. 4), we computed a misclassification rate (MR, Eq. 5) for every AMO in the test subset of each model, defined as the percentage of incorrect predictions in test cases with AMO equal or higher than a given cutoff (Eq. 5). A regression model was fit to this MR distribution for each drug and then was used to calculate the MR for new model outputs (Supplementary Figure S1). The RI is the inverse of the MR and simply defined as 1 – MR.

(4) $$A\,M\,O\,\left(x\right)=\left\{\begin{array}{cc} x & ,x\geq 0.5\\ 1-x & ,x<0.5 \end{array}\right\}$$

(5) $$M\,R\,\left(c\right)=\frac{\text{fp}+\text{fn}}{\text{tp}+\text{fp}+\text{tn}+\text{fn}}$$

where tp, fp, tn, and fp are the number of true positive, false positive, true negative, and false negative instances, respectively, predicted with AMO ≥ *c*.

### Feature Importance

XGBoost, being a random forest-based algorithm, can provide important features from the model once it has been built in order to evaluate the individual feature impact in the decision-making process. In our case, XGBoost lists the most important ARGC for each model. By design, random-forest-based algorithms ignore strongly correlated features while using only one in the model, as adding redundant features will not provide extra discrimination capabilities (see section “Building and evaluating machine learning models”). From a biology standpoint, however, it is interesting to know all the ARGC that occur in high frequency. Thus, when the most important features are extracted from the models, we reviewed the correlated features ARGC as well.

In consideration that organism(s)/strain(s) composition in the sample should not be known *a priori*, whereas *de novo* genome assembly may be inefficient and inaccurate, no genome assembly from WGS data is conducted in this work. Therefore, the prediction model is agnostic to the source of the detected antimicrobial gene, as to whether it is inherent to an organism or acquired via mobile genetic element (plasmid). Hence, no weighting scheme for plasmid-derived genes was considered for the model.

### Preliminary Pipeline Implementation and Evaluation

The long term goal of this project is to build a platform where prediction models like the ones presented here can be used in research or clinical practice to quickly estimate a bacterium’s antibiogram from WGS data with sufficient accuracy, in order to inform early decisions about the correct AB use while awaiting the final antibiogram.

For illustration of developed models, an R-Shiny web-based application was developed where pre-processed samples from two datasets unseen in training (31 public, demo dataset, and 19 in-house samples, sections “Public data collection” and “In-house dataset of samples”) can be individually submitted to the prediction models and subsequently compared to their actual ABR status. Furthermore, the application allows the user to explore the performance of the current ABR models in more detail, and review the important genes used in the decision-making process.

## Results

### Data Collection and Pre-processing

After the processing and filtering (sections “Analysis of WGS data” and “Clustering similar antibiotic resistance genes”), of the 4579 ARG in the NCBI database 2605 (57%) were detected in at least one of the 946 samples. When clustered, only 152 ARGC (15%) were detected in the whole dataset. The median number of ARGC present in any sample is 10 resulting in a very sparse dataset. Table 2 lists the most frequently found ARGC per species.

**TABLE 2:** Most common antibiotic resistance gene clusters per species detected from the WGS data.

**Species (Total samples)**	**Most common gene (% occurrence)**	**Second most common gene (% occurrence)**
*A. baumannii* (264)	Class C beta-lactamase ADC-98 (98.5)	OXA-51 family carbapenem-hydrolyzing class D beta-lactamase OXA-561 (98.5)
*E. coli* (341)	Class C extended-spectrum beta-lactamase EC-18 (99.4)	Aminoglycoside O-phosphotransferase APH(3″)-Ib (51.3)
*E. cloacae* (70)	Multidrug efflux RND transporter permease subunit OqxB21 (92.2)	Fosfomycin resistance glutathione transferase FosA2 (78.6)
*K. aerogenes* (53)	Multidrug efflux RND transporter permease subunit OqxB21 (100.0)	FosA family fosfomycin resistance glutathione transferase (100.0)
*K. pneumoniae* (218)	FosA family fosfomycin resistance glutathione transferase (100.0)	Class A beta-lactamase SHV-200 (96.3)

*The occurrence refers to the percentage of samples, stratified by species, where a given ARGC is found in the WGS data.

**FIGURE 2:**
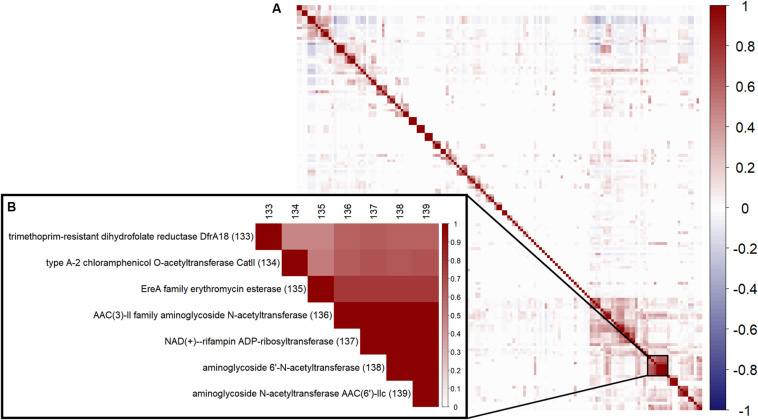
Correlation between the 152 ARGC found in the analyzed samples. **(A)** Overall pairwise correlation plot. **(B)** A zoom-in example of a highly correlated group of ARGC. Numbers in parentheses are the same serial numbers as in columns provided for easy matching.

To review correlation between the 152 ARGC, a hierarchical clustering using Ward’s algorithm (Ward, 1963) was performed (Figure 2A). An example of the strong correlation between several specific ARGC can be seen in Figure 2B. Even though some of the clusters have similar names, they represent different subtypes of the same gene (i.e., different enough in sequence to be placed in separate clusters, section “Clustering similar antibiotic resistance genes”). The full table with all ARGC pairwise correlation values can be found in supplements (Supplementary Table S4).

### XGBoost Model Training and Testing

**FIGURE 3:**
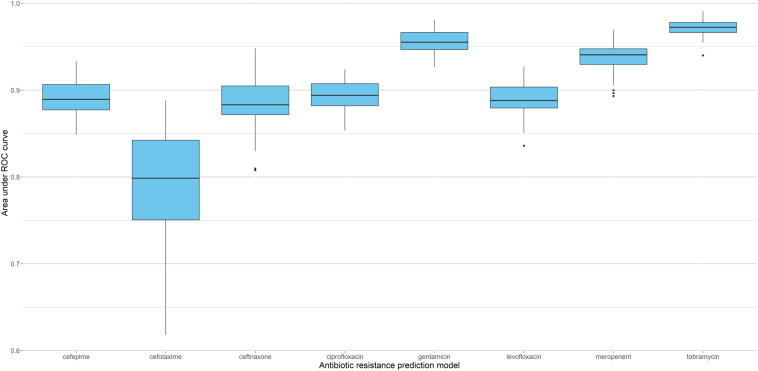
Performance of XGBoost models based on 51 different splits of the data. Boxplots represent distribution of AUC for the corresponding testing subsets, with thick lines indicating the median performance.

**FIGURE 4:**
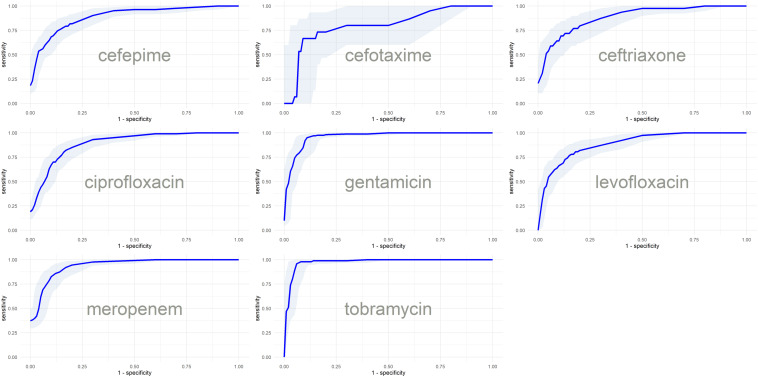
ROC curves with confidence intervals of the final models based on the predictions of test subsets.

Since sparse input in machine learning models can bias performance (both under- or overestimating) depending on the split in training and testing data (Wu et al., 2013), we trained 51 independent models for each antibiotic (polling isolates of all species together). Each model was trained and validated with a different split in order to see the performance distribution (refer to Supplementary Figure S2 for the overall flow of model training). Figure 3 shows the distribution of AUC based on the testing subset for each individual split. The model with the median performance over all 51 splits for each antibiotic (represented by the thick line in the boxplots) is assumed to be the closest to real-life performance (i.e., the least biased) and was chosen as the final model. The performance of these final models are detailed in Table 3 and Figure 4 (see Supplementary Table S5 for the performance of all other models).

**TABLE 3:** Performance of final ABR prediction models.

**Antibiotic**	**Acc**	***R***	***P***	**Sp**	**MCC**	**AUC**	**PR-AUC**
Cefepime	0.82	0.86	0.86	0.77	0.62	0.89	0.92
Cefotaxime	0.83	0.93	0.86	0.53	0.52	0.80	0.92
Ceftriaxone	0.84	0.93	0.87	0.56	0.55	0.88	0.96
Ciprofloxacin	0.81	0.86	0.85	0.73	0.60	0.89	0.94
Gentamicin	0.91	0.90	0.89	0.93	0.82	0.96	0.95
Levofloxacin	0.81	0.87	0.84	0.70	0.58	0.89	0.94
Meropenem	0.89	0.82	0.92	0.94	0.78	0.94	0.94
Tobramycin	0.95	0.92	0.98	0.98	0.90	0.97	0.98

### Feature Importance

Regardless of fluctuations in model performance based on split in training and testing, the important features ARGC extracted from the models appeared to be largely the same per antibiotic prediction. Table 4 shows the 5 most important ARGC (on average over the 51 models) for each ABR prediction model. The full table can be found in the supplements (Supplementary Table S6).

**TABLE 4:** Top 5 most important features for each antibiotic model.

**ARGC**	**Gain**
**Cefepime**	
AAC(6′)-Ib family aminoglycoside 6′-N-acetyltransferase	18.90 ± 3.27
Class A extended-spectrum beta-lactamase CTX-M-222	7.84 ± 1.07
Aminoglycoside O-phosphotransferase APH(3″)-Ib	6.44 ± 1.91
Class C extended-spectrum beta-lactamase EC-18	5.49 ± 1.30
Carbapenem-hydrolyzing class A beta-lactamase KPC-33	5.14 ± 1.32
**Cefotaxime**	
Aminoglycoside nucleotidyltransferase ANT(3″)-IIa	17.09 ± 7.27
AAC(6′)-Ib family aminoglycoside 6′-N-acetyltransferase	13.75 ± 7.65
Class C extended-spectrum beta-lactamase EC-18	13.16 ± 4.84
Multidrug efflux RND transporter permease subunit OqxB21	9.74 ± 4.27
OXA-51 family carbapenem-hydrolyzing class D beta-lactamase OXA-561	8.26 ± 3.86
**Ceftriaxone**	
AAC(6′)-Ib family aminoglycoside 6′-N-acetyltransferase	15.52 ± 4.35
Class C beta-lactamase CMY-163	8.76 ± 1.79
Class A extended-spectrum beta-lactamase CTX-M-222	8.37 ± 2.17
Class C extended-spectrum beta-lactamase EC-18	8.10 ± 1.99
Multidrug efflux RND transporter permease subunit OqxB21	7.00 ± 3.20
**Ciprofloxacin**	
AAC(6′)-Ib family aminoglycoside 6′-N-acetyltransferase	23.44 ± 4.94
Sulfonamide-resistant dihydropteroate synthase Sul1	10.29 ± 3.20
Tetracycline efflux MFS transporter Tet(B)	4.99 ± 1.71
Class A beta-lactamase TEM-219	4.93 ± 1.14
Aminoglycoside O-phosphotransferase APH(3″)-Ib	4.47 ± 1.63
**Gentamicin**	
Aminoglycoside N-acetyltransferase AAC(3)-IIc	28.79 ± 3.10
ANT(3″)-Ia family aminoglycoside nucleotidyltransferase AadA1	20.98 ± 2.52
Aminoglycoside nucleotidyltransferase ANT(2”)-Ia	17.79 ± 2.03
OXA-24 family carbapenem-hydrolyzing class D beta-lactamase OXA-25	4.15 ± 1.59
Mph(E) family macrolide 2’-phosphotransferase	2.07 ± 1.10
Levofloxacin	
AAC(6′)-Ib family aminoglycoside 6′-N-acetyltransferase	25.11 ± 6.17
Sulfonamide-resistant dihydropteroate synthase Sul1	7.72 ± 3.73
Tetracycline efflux MFS transporter Tet(B)	5.92 ± 1.67
Class A beta-lactamase TEM-219	5.89 ± 1.74
Class C extended-spectrum beta-lactamase EC-18	4.97 ± 1.73
**Meropenem**	
Carbapenem-hydrolyzing class A beta-lactamase KPC-33	30.09 ± 5.21
Bleomycin binding protein Ble-MBL	9.55 ± 2.02
OXA-23 family carbapenem-hydrolyzing class D beta-lactamase OXA-483	8.58 ± 3.25
Class C extended-spectrum beta-lactamase EC-18	5.79 ± 2.05
Class A beta-lactamase SHV-200	5.59 ± 2.74
**Tobramycin**	
AAC(6′)-Ib family aminoglycoside 6′-N-acetyltransferase	60.50 ± 5.02
Aminoglycoside nucleotidyltransferase ANT(2”)-Ia	17.91 ± 1.98
Aminoglycoside N-acetyltransferase AAC(3)-IIc	6.54 ± 1.24
Aminoglycoside 6′-N-acetyltransferase AAC(6′)-Iq	4.62 ± 1.51
ArmA family 16S rRNA [guanine(1405)-N(7)]-methyltransferase	1.89 ± 0.97

*Gain is the relative importance of the ARGC in a prediction model, reported here as mean with standard deviation computed over 51 independent models for the same AB.

As mentioned in section “Feature importance”, once a feature is chosen for the use in the decision-making, random forest-based methods often ignore other highly correlated features as they do not contribute to class discrimination. However, in the context of this study, when unused ARGC may provide additional biological insights, we list all ARGC, chosen by the models and those correlated, in supplemental materials (Supplementary Table S4).

### Comparison With Other Algorithms

XGBoost models were compared with those based on LASSO and Ridge regression, which also can deal with data sparsity and have built-in feature selection (Supplementary Table S7). In 6–7 (depending on the metric to compare) out of 8 AB, XGBoost models appear to be slightly better than linear regression models. The real advantage of XGBoost over the linear regression algorithms is the robustness in the important feature selection. Table 5 in conjunction with Supplementary Table S6 demonstrate that XGBoost yields the best consistency in selecting top informative features compared to LASSO and Ridge regression models.

**TABLE 5:** Counts of unique features found among top 5 across 51 independent models for each AB.

**Antibiotic model**	**LASSO**	**Ridge**	**XGBoost**
Cefepime	28	13	11
Cefotaxime	38	38	12
Ceftriaxone	25	29	12
Ciprofloxacin	30	16	13
Gentamicin	16	12	18
Levofloxacin	24	17	11
Meropenem	25	18	11
Tobramycin	14	16	14

*The lower number signifies the more consistent feature selection across data resampling.

### Reliability Indexes

**FIGURE 5:**
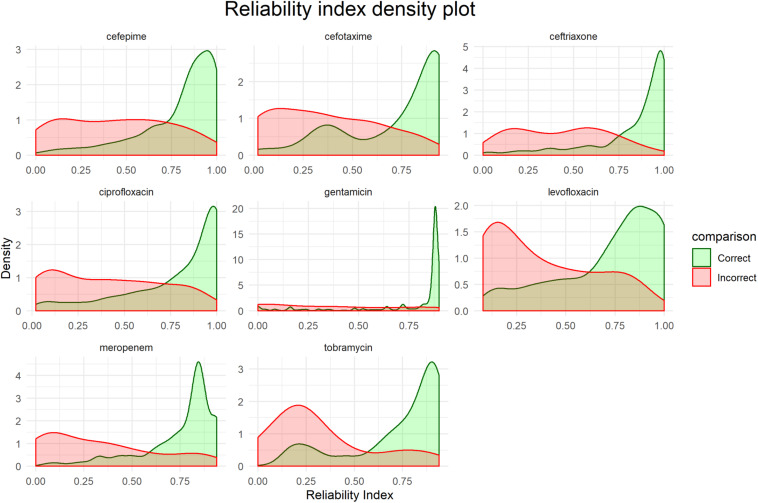
Distribution of reliability indexes (RI). Density plots are based on the predictions of the respective testing sets for each final model.

Figure 5 shows distributions of the RI for each final model based on the corresponding testing set. While there is no unified cut-off for RI across all models, there is a clear trend that correct predictions tend to have a higher RI.

### Practical Implementation of Models

**FIGURE 6:**
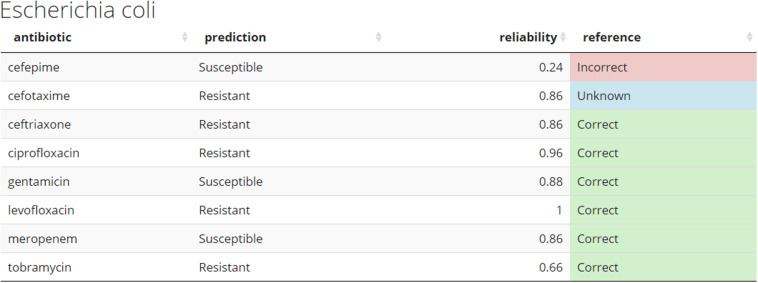
Example of the predicted antibiogram. An illustration of the output from the *R* shiny app using the sample SAMN07450853 from the Demo set. Other samples from the Demo and In-house datasets can be accessed through the app. The “reference” column compares the predicted resistance to the one confirmed in the clinical laboratory. This would normally not be present at the time the models do their prediction for *de novo* samples. Color coding used: green – correct, red – incorrect, and blue – unknown.

Demo dataset (section “Preliminary pipeline implementation and evaluation”) is used to illustrate how new unseen samples could be run through the pipeline of preprocessing and subsequent prediction by the 8 models (Figure 6). For a given sample, each antibiotic is assigned a binary resistance prediction with a confidence (reliability index). For demonstration purposes, current implementation retrieves meta-data information for a given sample, such as species name (a header of the table), and known drug resistance status (last colored column).

**FIGURE 7:**
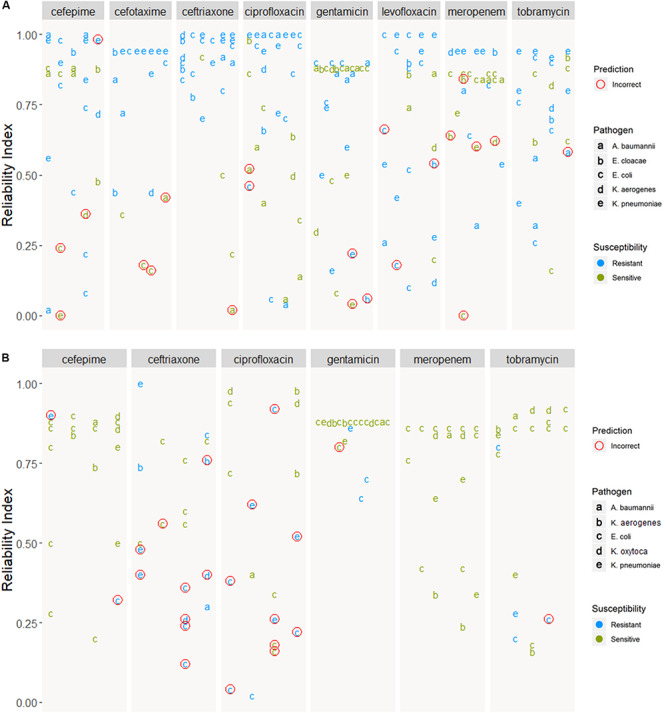
Prediction of samples from the **(A)** Demo and **(B)** In-house datasets. Predictions are grouped by antibiotic and ordered by reliability index. Incorrect predictions encircled in red, predictions with no known resistance in the meta-data are not shown. In-house dataset was not tested for cefotaxime and levofloxacin, hence these two ABs are not shown.

Figure 6 shows how prediction results on novel samples could be presented to the clinician in the format of an antibiogram. The online application provides a more intuitive way to explore the results and use of this pipeline. The predicted antibiograms from all extra samples (the Demo set based on public samples and the In-house dataset) can be explored in detail, and an additional tab (not shown) provides more information about the models and the important features. The summary of all predictions for Demo and In-house datasets can be found in Figure 7. The web-based demonstration and stand-alone versions of the application can be found at https://vancampn.shinyapps.io/wgs2amr/ and https://github.com/pieterjanvc/wgs2amr/, respectively.

## Discussion

This work provides a framework wherein bacterial samples can be tested quickly to obtain a preliminary antibiogram to guide initial antibiotic selection for treatment. Culturing bacteria and getting a full antibiogram can take 2–4 days, whereas WGS and the computational pipeline presented here only takes around a day. At present, sequencing is taking up the majority of time but is likely to decrease significantly with the improvements in sequencing technologies.

Presenting the results as an early antibiogram estimate (Figure 6) instead of just individual predictions, provides clinicians with a clear and intuitive way to inform the choice of the otherwise empiric initial AB. The general resistance pattern of the whole antibiogram can be informative in itself, even if there may still be errors in individual predictions. The latter is further aided by the addition of the RI that indicates how certain the models are on individual predictions. While there is not a single clear cut for the RI, Figures 5, 7 suggest that the majority of samples assessed with a high RI appear to have correct predictions. All of this helps early, informed AB choice that can decrease the time to start effective AB therapy while limiting the use of empiric broad-spectrum AB and slowing the development of new resistant strains. The predictions could become especially helpful in settings where resources do not permit the use of a full microbiology lab (e.g., in developing countries). Upcoming technologies, like the Nanopore MinION, (Z) (2019), will allow clinicians in the near future to sequence pathogens with smaller portable devices. This, coupled with analytical pipelines like the one presented here, could provide valuable information on pathogen resistance that would otherwise not be available. Regardless, the results will require clinical judgment.

All pathogens have several ARGC that are found in nearly every sample, regardless of its resistance status to the tested AB (e.g., *class C extended-spectrum beta-lactamase EC-18* cluster was detected in 99% of *E. coli* isolates, Table 2). This underlines that Gram-negatives have no easy one-to-one genotype-phenotype relationship for some ARG as their presence does not equal resistance *per se*. A well-studied example of this is the *AmpC* gene, which is expressed in many species or strains, even those fully susceptible to AB (Bajaj et al., 2016). The complex relationships between the ARG and phenotype dictated the application of more complex machine learning algorithms, such as random forest (the basis of XGBoost). An additional advantage of XGBoost is that it provides a glimpse into its decision-making process by reporting the list of features ARGC most often used when building the model as a proxy for key decisions. The downside of this simplification is that caution is warranted when interpreting these features. Finally, given that the datasets are sparse, models are prone to having to rely on different features depending on the split in training and testing. The higher the consistency in selecting important features across different independent models, the more robust the final model is anticipated to be (Table 5).

An intuitive example is the *class A extended-spectrum beta-lactamase* cluster as an important feature in the cephalosporin prediction models (Table 4). Other ARGCs seem less relevant at first glance but might make more sense when interpreted in a broader context. *AAC(6′)-Ib family aminoglycoside 6′-N-acetyltransferase* is an aminoglycoside resistance gene, but apart from being important in the tobramycin model (an aminoglycoside), it is also found to be important in the models predicting cephalosporin and levofloxacin resistance. This is where a more careful interpretation of important features is warranted as the non-linearity of XGBoost models is starting to provide less intuitive connections. For example, this gene is found in a variety of Gram-negative species and is known to be present in many different plasmids, genomic islands and integrons that carry other genetic resistance genes. It could be that this gene is a proxy for other, more relevant genes or even other genetic factors in the genome associated with resistance (Wilson et al., 2016; Lehtinen et al., 2017). Furthermore, this gene is prominent in *Klebsiella* species which could be used during decision-making to take advantage of innate differences in resistance between species. The fact that our prediction models are species independent could make them more powerful when focusing on resistance patterns in contaminated, mixed, and metagenomic samples. The latter is part of the future goals of this work. To show that the presence of ARG only could easily predict species, a model with the same input, but trained on predicting species instead of ABR, was built and had a near perfect accuracy (Supplementary Table S8).

The most striking discordance between an antibiotic and its model’s important ARGC features was observed for levofloxacin. None of the most important ARGC picked up by the model are directly related to quinolone resistance. This is likely because levofloxacin resistance is largely based on mutations or small variations (e.g., gyrase gene; Chen and Lo, 2003). Given our models do not incorporate such information (section “Clustering similar antibiotic resistance genes”), all important ARGC in the levofloxacin resistance model appear to be proxies for these mutations. This illustrates both the strength and limitations of models like XGBoost. It can make accurate predictions (Table 3) on correlated and complex data by using non-linear logic, but the interpretation of such models can be obscure and could limit biological understanding of the underlying processes.

As any other previous work in this early-stage field of predicting ABR based on the WGS data, our study has several limitations. One of the main challenges was the sparsity of the model input, which may result in biased performance depending on the split in training and testing data. Even after clustering highly similar ARG in ARGC (hence no account for polymorphism), we still ended up with some ARGC only seen once in the whole dataset (median presence of 10 out of 152 clusters per sample). The sparsity is likely because some genes are rare, or the dataset is not fully representative of all evaluated resistances (limitation of using publicly available data). If we would only have used the genes originally sequenced in the bacteria of interest, we might have had less sparsity, but would likely be underestimating the presence of resistance as many species have developed similar resistance genes or exchanged them in processes like horizontal gene transfer. The second reason performance suffered in some cases is the class imbalance between available susceptible and resistant samples, e.g., cefotaxime only has 50 susceptible samples (Table 1), and also the lowest performance (Table 3). By creating many independent models for each antibiotic and selecting the one with median performance, we ensured that the final model would be the closest estimate of the real-life performance (section “XGBoost model training and testing”). This technique is not to be confused with model cross-validation, where different splits of data are used to enhance one final model, and additional validation data is needed to estimate the performance.

Other limitations are that the models cannot predict the level of resistance (i.e., as regression) as they were trained on a binary data (resistant versus susceptible). Using the MIC values as input could help in this case, but the data is not always available or may be inconsistent. Also, the current predictor accounts for presence or absence of certain known drug resistance genes and hence cannot detect the presence of previously unseen genes conferring new drug resistance. In other words, the prediction of a sample being resistant has higher confidence than being susceptible. Furthermore, due to the nature of sequencing data (DNA-seq), the models cannot incorporate resistance originating from the over-expression of genes targeted by inhibitors. RNA-seq potentially could mitigate this problem but presents additional challenges. (Meta-)transcriptomics data, unfortunately, and remains mostly within research realm. Complexity of reference database, inference of organisms/strains, and their relative abundance data (Cox et al., 2017), dynamic gene expression profile upon different drug treatments, and overall complexity of data preparation/generation impede the application of meta-transcriptomics data for real-time predictions (Van Camp et al., 2020).

Finally, the models use sequencing data derived from isolates, but it remains to be seen how they would perform on contaminated or mixed (e.g., metagenomic) samples. All limitations will be further addressed in future studies. Nevertheless, this study has shown a great potential of sequencing data as a basis for the prediction of antimicrobial resistance in Gram-negative bacteria. We additionally focused on the more practical implementation of resistance prediction models by presenting the end-users (medical practitioners) with an easy to use and interpret interface where novel predictions on different AB are shown together as in a traditional antibiogram but with an additional RI to further assist during the decision making process.

To summarize, this work demonstrates that whole-genome sequencing coupled with modern machine-learning methods has great potential to deliver early estimations of the antibiogram for Gram-negative bacteria. The generated models, being trained solely on the presence-absence of the clusters of ARG, demonstrate promising performance and robustness to heavily class-imbalanced data. RI are introduced to provide further assessment of predictions and may be used in subsequent machine learning models to improve accuracy further. By presenting the results in the form of an antibiogram, we provide an intuitive way for the clinician to interpret predictions and guide the initial empiric antibiotic choice before the laboratory results are available. This may help in shortening the time to start effective AB treatment.

## Data Availability Statement

The datasets generated for this study can be found in the NCBI BioSample database (BioProject ID: PRJNA587095).

## Author Contributions

P-JV did the data collection and curation, model training and validation, developed the R shiny app, and stand-alone analytical pipeline. DH and AP conceived of the study, evaluated results, and supervised the project. DH provided clinical samples for the In-house dataset. AP provided computational resources. All authors participated in writing the manuscript.

## Conflict of Interest

The authors declare that the research was conducted in the absence of any commercial or financial relationships that could be construed as a potential conflict of interest.

## Abbreviations

AB: antibiotic
ABR: antibiotic resistance
ARG: antibiotic resistance gene
ARGC: antibiotic resistance gene cluster
MCC: Matthew’s correlation coefficient
MIC: minimum inhibitory concentration
WGS: whole genome sequencing.
