# Supplementary material for: Prediction of Antimicrobial Resistance in Gram-Negative Bacteria From Whole-Genome Sequencing Data
Source: Front Microbiol. 2020 May 25;11:1013. doi: 10.3389/fmicb.2020.01013 (PMC7262952; doi:10.3389/fmicb.2020.01013)
Supplement: Supplementary file 1 [file Image_1.PDF]

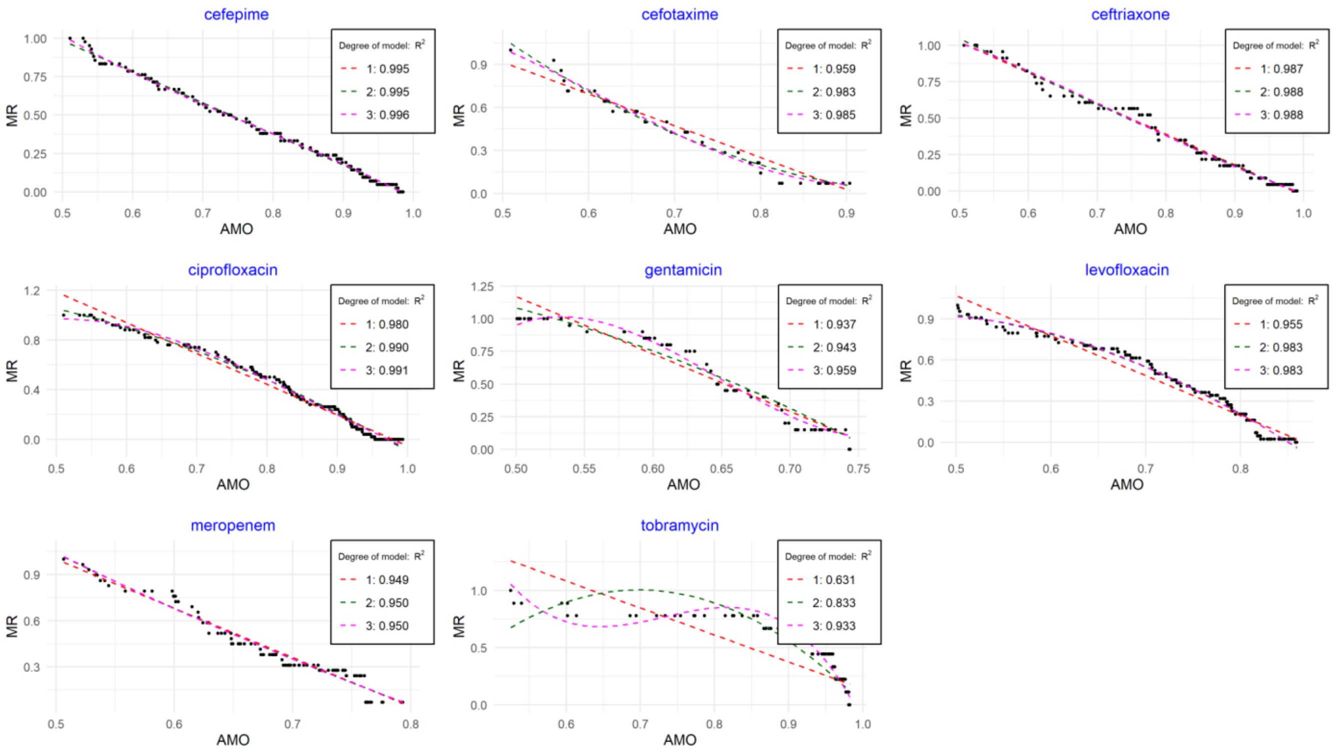

Figure S1. Calibration plots for reliability indexes

Different polynomial fits for the misclassification rate (MR) versus the adjusted model output (AMO) with corresponding R^2 values were computed. The third-degree polynomial was chosen as best fit and was used to calculate the reliability index (defined as 1 – MR) AMO of new samples (used in Demo and In-house dataset).
