# Supplementary material for: Prediction of Antimicrobial Resistance in Gram-Negative Bacteria From Whole-Genome Sequencing Data
Source: Front Microbiol. 2020 May 25;11:1013. doi: 10.3389/fmicb.2020.01013 (PMC7262952; doi:10.3389/fmicb.2020.01013)
Supplement: Supplementary file 2 [file Image_2.PDF]

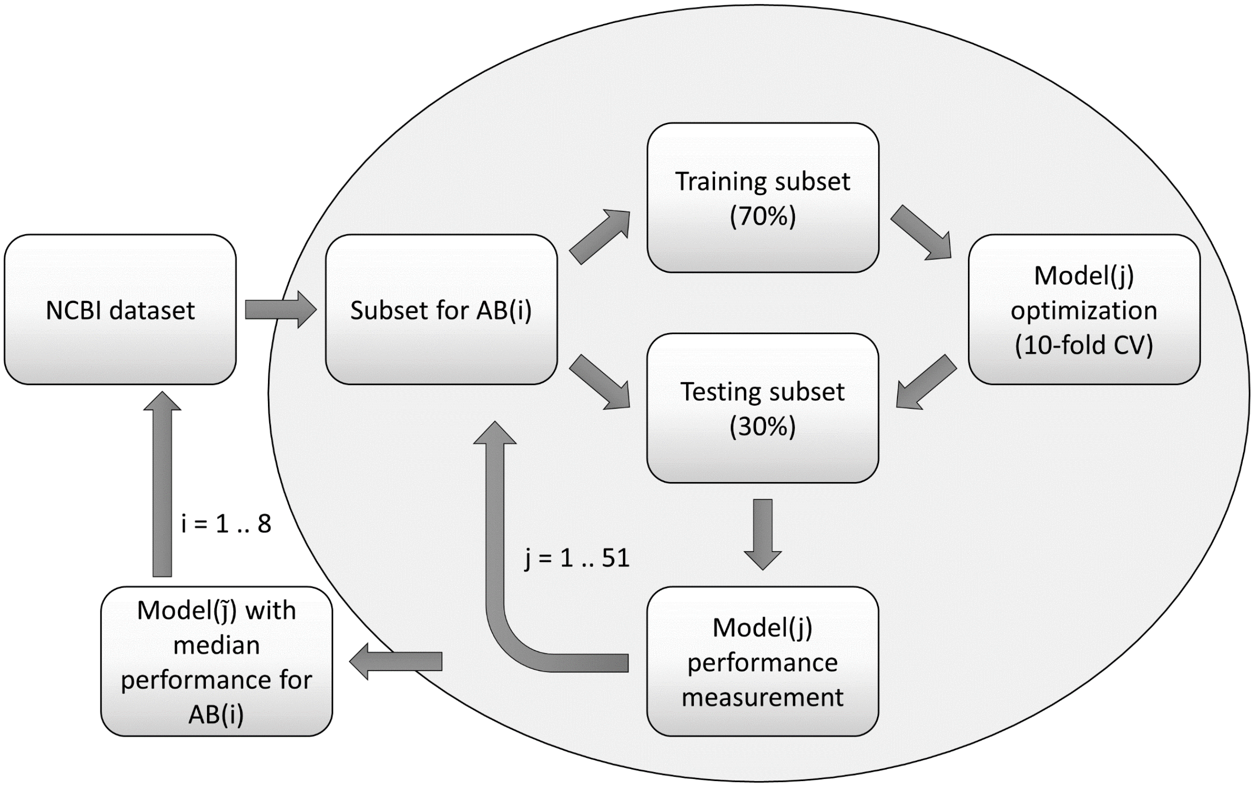

Figure S2. The machine learning flow used to build eight models based on sparse data

For each of eight drugs 51 random data splits were performed. Training subset (70%) was used in building the model (optimized with XGBoost 10-fold cross-validation), whereas testing subset (30%) was used to evaluate the resulting model. Median performing model (26th) was chosen as a final prediction model for each drug.
